# Endoscopic ultrasound-guided left gastric vein embolization: a novel minimally invasive approach for gastroesophageal varices-1

**DOI:** 10.1055/a-2975-3253

**Published:** 2026-10-16

**Authors:** Guilian Cheng, Jialiang Huang, Duanmin Hu

**Affiliations:** 1 Department of Gastroenterology 105860 Second Affiliated Hospital of Soochow UniversitySuzhouJiangsuChina

Gastroesophageal varices-1 (GOV1) is the most prevalent subtype in clinical practice, manifesting as esophageal varices extending down to cardia or lesser curvature. Guidelines recommend endoscopic variceal ligation or endoscopic sclerotherapy injection, yet these modalities can only obliterate superficial mucosal varicose vessels and fail to occlude the primary feeding branches outside the gastric wall, leading to a high recurrence rate. 1​ Alhough percutaneous transhepatic variceal embolization enables embolization of the left gastric vein (LGV), it requires traversing hepatic parenchyma and the portal vein, carrying substantial risks. In this case, we integrated endoscopic and interventional therapeutic concepts and pioneered an endoscopic ultrasound–guided left gastric vein embolization (EUS-LGVE).

A 61-year-old female patient with liver cirrhosis presented to our hospital with recurrent melena. Endoscopy revealed multiple tumor-like varices in the middle and lower esophagus extending continuously to the posterior wall of the cardia, with no varices observed in the gastric fundus ( Fig. 1 ), consistent with GOV1. Portal vein computed tomography venography clearly delineates the hemodynamic profile of the varices, revealing the LGV arising from the main trunk of the splenic vein ( Fig. 2 ). After multidisciplinary evaluation, EUS-LGVE was selected as the definitive treatment ( Video 1 ). First, we located the splenic vein under EUS and identified the LGV branching off the main trunk of the splenic vein ( Fig. 3a ). With the LGV as the reference landmark, a scan along the lesser gastric curvature toward the cardia. The LGV was observed to give off multiple branches, among which the anterior branch was the thickest, with an internal diameter of approximately 4 mm. This branch extended continuously into the submucosa of the cardia and served as the vascular origin of esophageal varices. Subsequently, under EUS guidance, the segment of the anterior branch proximal to the main trunk of the LGV was selected as the puncture site. The sandwich technique, combining coil deployment and tissue adhesive injection, was performed with a 22-gauge needle, resulting in the successful embolization of the LGV ( Fig. 3b ). The tissue adhesive used in our center is n‑butyl‑2‑cyanoacrylate, which is applied without mixing with lipiodol. The injection technique was performed as follows: after one coil (8×12 mm) deployment, the injection needle was primed with lauromacrogol. A total of 0.5 mL of lauromacrogol was first injected as a tracer, followed by rapid injection of 1 mL of n‑butyl‑2‑cyanoacrylate, and finally another 0.5‑ml lauromacrogol was injected. Repeated EUS scanning demonstrated that the native luminal structure of the main LGV trunk was no longer identifiable, and its distal branches were completely replaced by hyperechoic glue casts ( Fig. 3c ). White-light endoscopy confirmed collapse of the esophageal varices ( Fig. 4 ). Antibiotics were administered both before and after the procedure. No adverse events occurred during or after the procedure. Postoperatively, the patient’s stool changed from black melena to yellow, with a recovery in hemoglobin levels.

**Fig. 1:**
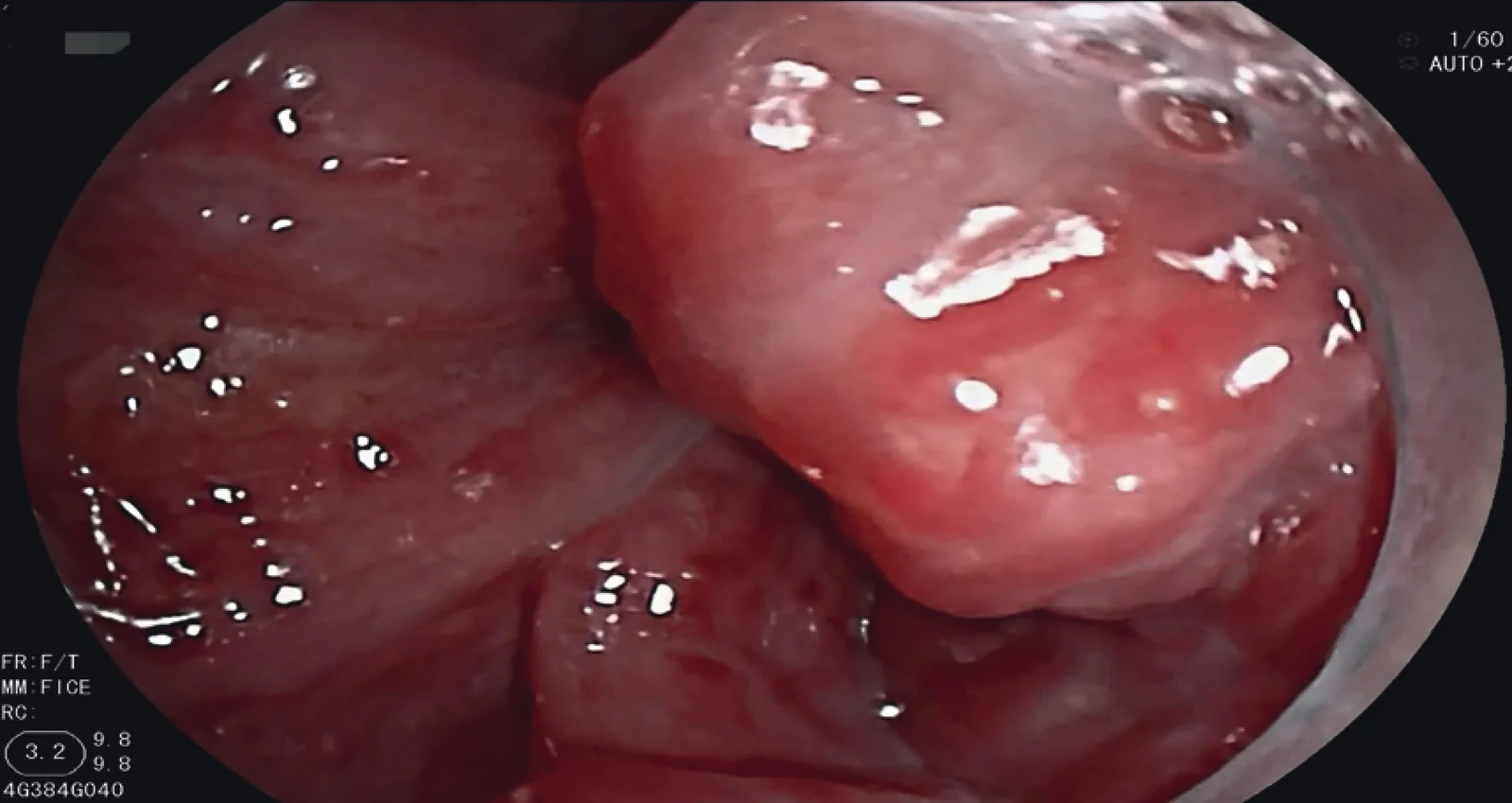
Pre-EUS-LGVE endoscopy: severe esophageal varices.

**Fig. 2:**
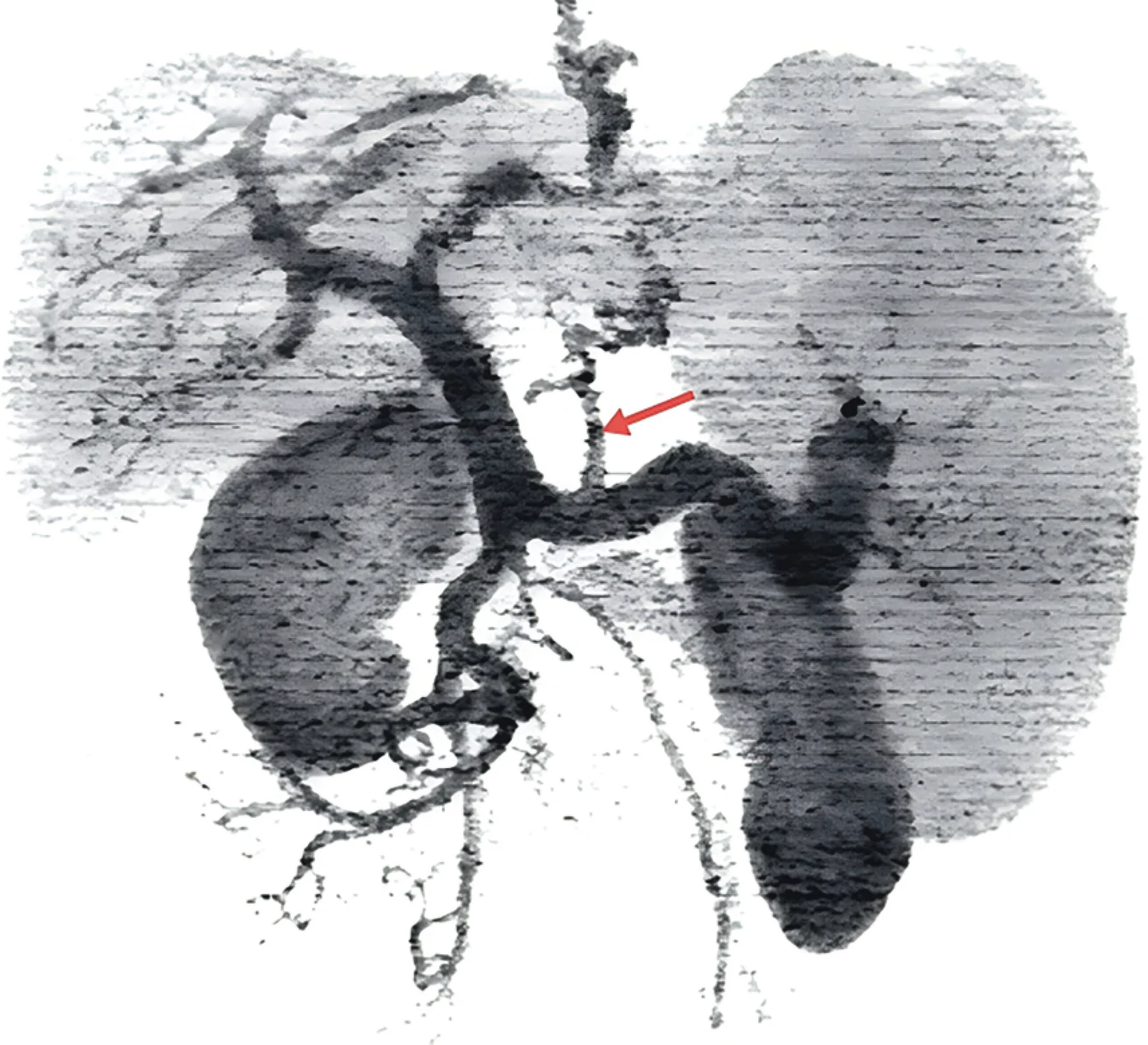
Portal venous CT venography images: The LGV (red arrow) arised from the main splenic vein trunk and extended into the submucosa of the cardia.

**Video 1** EUS-guided left gastric vein embolization with combined coils and tissue adhesive.


**Fig. 3:**
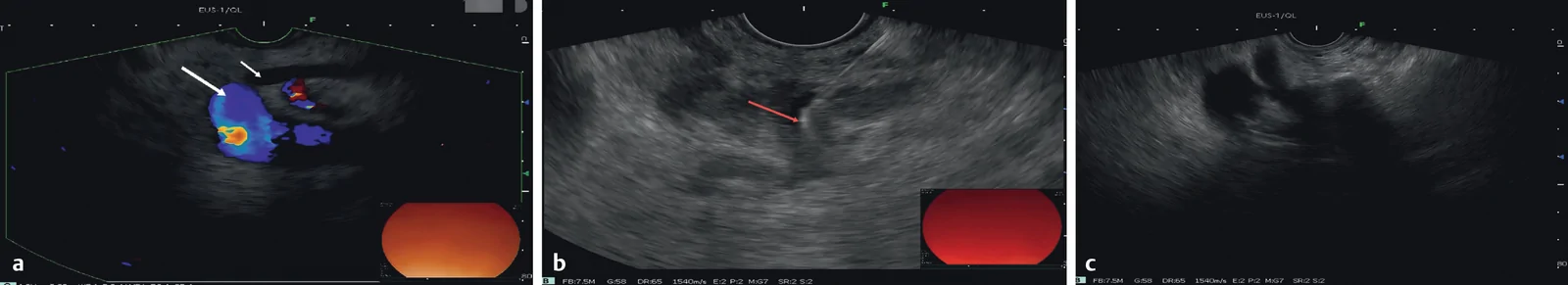
EUS-LGVE ( **a** ) EUS identification of LGV (short arrow) arising from the splenic vein trunk (long arrow). ( **b** ) Coils (red arrow) were deployed into the LGV through the tip of needle. ( **c** ) Post EUS-LGVE showing the loss of native LGV trunk lumen and replacement of distal branches by hyperechoic glue casts.

**Fig. 4:**
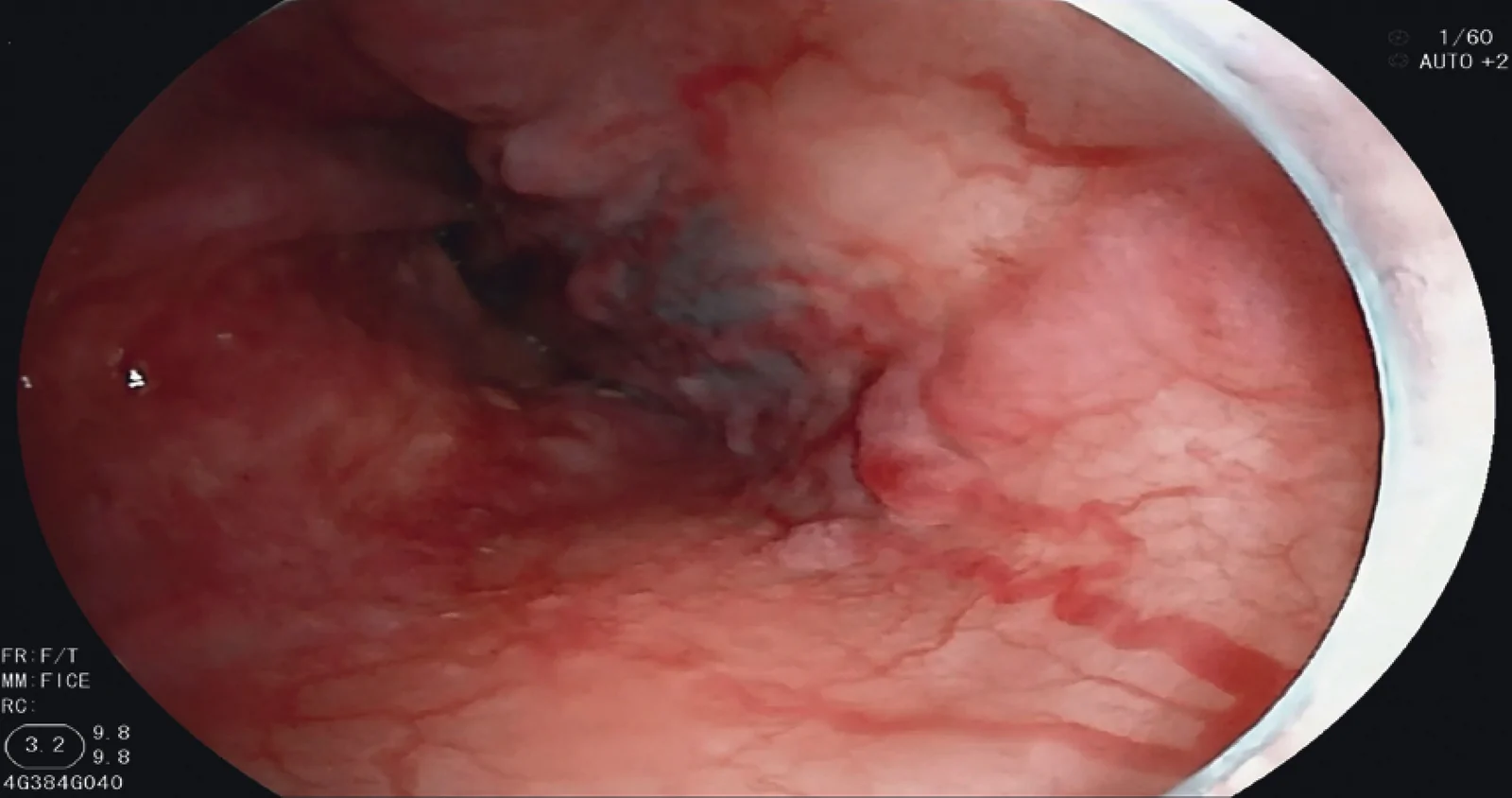
Post EUS-LGVE endoscopy: marked collapse of esophageal varices.

EUS-LGVE is a novel minimally invasive EUS-based interventional technique developed by our team. This technique not only enables continuous real-time tracking of the LGV and precise embolization of the target vessel throughout the procedure, but also allows immediate confirmation of therapeutic efficacy via two modalities: white-light endoscopy and EUS. This method can fundamentally reduce venous wall tension in GOV1 and eliminate the hemodynamic driver of variceal bleeding.

Endoscopy_UCTN_Code_TTT_1AS_2AL

## Clinical Trial Registration

This is a single clinical case report, not a prospective interventional clinical trial. Therefore, clinical trial registration is not applicable and not required.

## Declaration of GenAI Use

The authors confirm that no generative artificial intelligence was used in the preparation of this manuscript.
